# Community point distribution of insecticide-treated bed nets and community health worker hang-up visits in rural Zambia: a decision-focused evaluation

**DOI:** 10.1186/s12936-016-1165-1

**Published:** 2016-03-03

**Authors:** Paul Wang, Alison L. Connor, Ammar S. Joudeh, Jeffrey Steinberg, Ketty Ndhlovu, Musanda Siyolwe, Bristol Ntebeka, Benjamin Chibuye, Busiku Hamainza

**Affiliations:** IDinsight, 23B Twin Palm Road, Lusaka, Zambia; IDinsight, ADRI 1st Floor, BSIDC Colony, Off Boring Rd., Patna, Bihar 800013 India; Ministry of Health, National Malaria Control Centre, Lusaka, Zambia; Rufunsa District Health Office, Rufunsa, Zambia; World Vision, Plot No. 51/52, Great East Road, P.O. Box 31083, Lusaka, Zambia; Clinton Health Access Initiative, 175 Kudu Road, Lusaka, Zambia

**Keywords:** Malaria prevention, Insecticide-treated bed nets, Community point distribution

## Abstract

**Background:**

In 2013, the Zambian Ministry of Health through its National Malaria Control Programme distributed over two million insecticide-treated bed nets (ITNs) in four provinces using a door-to-door distribution strategy, and more than 6 million ITNs were allocated to be distributed in 2014. This study was commissioned to measure attendance rates at a community point distribution and to examine the impact of follow-up community health worker (CHW) hang-up visits on short and medium-term ITN retention and usage with a view of informing optimal ITN distribution strategy in Zambia.

**Methods:**

Households received ITNs at community point distributions conducted in three rural communities in Rufunsa District, Zambia. Households were then randomly allocated into five groups to receive CHW visits to hang any unhung ITNs at different intervals: 1–3, 5–7, 10–12, 15–17 days, and no hang-up visit. Follow-up surveys were conducted among all households at 7–11 weeks after distribution and at 5–6 months after distribution to measure short- and medium-term household retention and usage of ITNs.

**Results:**

Of the 560 pre-registered households, 540 (96.4 %) attended the community point distribution. Self-installation of ITNs by households increased over the first 10 days after the community point distribution. Retention levels remained high over time with 90.2 % of distributed ITNs still in the household at 7–11 weeks and 85.7 % at 5–6 months. Retention did not differ between households that received a CHW visit and those that did not. At 7–11 weeks, households had an average of 73.8 % of sleeping spaces covered compared to 80.3 % at 5–6 months. On average, 65.6 % of distributed ITNs were hanging at 7–11 weeks compared to 63.1 % at 5–6 months. While a CHW hang-up visit was associated with increased usage at 7–11 weeks, this difference was no longer apparent at 5–6 months.

**Conclusions:**

This evaluation revealed that (1) the community point distributions achieved high attendance rates followed by acceptable rates of short-term and medium-term ITN retention and usage, as compared to reported rates achieved by door-to-door distributions in the recent past, (2) CHW hang-up visits had a modest short-term impact on ITN usage but no medium-term effect, and (3) community point distributions can yield sizeable time savings compared to door-to-door distributions.

## Background

Despite improved malaria prevention and treatment efforts over the last decade, malaria remains one of Zambia’s greatest public health challenges with more than four million clinically diagnosed cases in 2010 [1, 2]. Malaria is endemic to every province in Zambia and contributes to 36 % of hospitalizations and outpatient visits, 8–14 % of low birth weight babies, 3–8 % of all infant deaths, and up to 20 % of maternal deaths [2, 3]. In 2013, more than 3500 Zambians died from malaria, and it is the leading cause of child mortality nationally [4, 5]. Additionally, malaria has serious economic implications from increased healthcare expenditures and decreased worker productivity [6].

Consistently sleeping under an insecticide-treated bed net (ITN) has been shown to decrease all-cause child mortality by 17 % and the frequency of severe malaria by 45 % [7]. Larger community-wide gains can be realized if a critical number of households use ITNs [8]. ITN coverage in Zambia has greatly increased in recent years, with 68 % of households owning at least one ITN in 2012 compared to 38 % in 2006 [2]. Despite these gains, many Zambians still remain to benefit from increased ITN ownership and usage.

Zambia was allocated approximately six million ITNs for immediate distribution in 2014, a sufficient number to cover every sleeping space nationwide. Establishing an effective and cost-effective strategy for distribution that achieved high retention and usage at the household level was critical to maximizing the benefits of these ITNs. Prior to 2014, national guidelines only allowed for door-to-door distributions, in which CHWs delivered and hung ITNs at every household. While this strategy was effective in some contexts, it was also burdensome and costly in many rural locations [9].

Zambia’s Ministry of Health (MOH) through its National Malaria Control Centre (NMCC) commissioned this study to inform the 2014 ITN distribution and beyond. Their first question was whether community point distributions could be a viable alternative to door-to-door distributions for reaching the majority of households. Free distribution of ITNs has been shown to be the most effective way to ensure the broadest distribution and the most lives saved with little difference in cost-effectiveness compared to cost-sharing mechanisms and direct purchase [10, 11]. Mass distributions can achieve high and equitable coverage in a short timeframe with continued retention and usage after the campaigns [12–15]. *Retention* is defined as ITNs remaining in the household to which they were distributed regardless of their being used, whereas *usage* is defined as household members sleeping under an ITN. While community point distributions have been used in other settings, they have not yet been tested in the Zambian context.

A second component of the current Zambian ITN distribution strategy relies on CHWs to hang all ITNs. Evidence is mixed, however, on whether a CHW visit influences ITN usage and retention. A non-randomised study from Ghana found that children in households where some or all ITNs had been hung by community health workers (CHWs) were more likely to sleep under an ITN than children from households that did not have any ITNs hung by a CHW [16]. Cluster randomised trials from Togo and Uganda, on the other hand, found that one or more CHW visits had limited to no detectable impact on ITN usage [17, 18].

This study sought to inform Zambia’s 2014 ITN distribution by examining three primary objectives: (1) Measure attendance at community point distributions in three rural communities in Zambia; (2) Measure self-installation rates of ITNs by households to examine cost-savings of delaying CHW visits; and (3) Estimate whether CHW visits are associated with higher short- and medium-term ITN retention and usage in rural Zambia. Since the study was to inform national policy, the study team also compared human resource needs among a community point distribution with a hang-up visit, a community point distribution with no hang-up visit, and a door-to-door distribution.

This study was conducted as part of the Demand-Driven Evaluations for Decisions (3DE) initiative. This initiative was implemented by the Clinton Health Access Initiative (CHAI) and IDinsight and funded by the UK’s Department for International Development (DFID) to conduct quick, low-cost, but rigorous evaluations to directly inform decisions identified by the Government of Zambia. World Vision provided the ITNs and preparatory logistical support before the study started. This study was designed and conducted in close collaboration with government stakeholders to ensure that the evidence would be actionable and applicable to the policy setting.

## Methods

### Study setting and population

This study was conducted between November 2013 and May 2014 in the three neighbourhood zones of Mukonka, Chipeketi, and Lukwipa in rural Rufunsa District, Zambia, to utilize an ITN distribution that was already planned to take place. Rufunsa District is a rural district in Lusaka Province where subsistence farming is the primary economic activity. The specific sites were chosen in consultation with the Rufunsa District Health Office (DHO) and rural health centre (RHC) staff to encompass rural communities with varying distances from the nearest RHC and from the major paved road that runs through Rufunsa District. The primary sample consisted of all households in these areas, which had been pre-registered by the NMCC in anticipation of standard door-to-door ITN distributions. Households that were missed during registration activities, but attended community point distributions were also included in the sample and received follow-up visits for a sensitivity analysis but were not included in the randomisation nor in the primary analyses.

### Study design and intervention

This study followed households that attended or were registered to attend a community point distribution over time. In addition to a cross-sectional measurement of community point distribution attendance, it used a randomised controlled trial design to assess the impact of a CHW hang-up visit on study outcomes. The intervention examined in this study consisted of (1) a community point distribution of ITNs and (2) a hang-up visit by a CHW. Since this study was commissioned to directly inform national policy, the intervention was implemented as closely as possible to what it likely would be at scale. This meant that community point distribution activities and CHW hang-up visits were conducted within existing distribution structures. Minimal support from study staff was provided to train CHWs on the conduct of the community point distribution and on establishing the optimal date for the distribution event. Distribution events were conducted at the neighbourhood zone level, ensuring that no beneficiary had to travel more than 4 h to attend a distribution. CHWs were instructed to use any available communication channels (i.e. schools, community events, markets) to inform households to collect their ITNs at the specified time and place.

On the day of distribution, household representatives gathered at the distribution site and received a health talk from CHWs emphasizing the importance of malaria prevention and ITN usage. Households were then individually called forward by name as a community verification mechanism. Since all attendees could hear which household was being called, it was difficult for someone other than a genuine household representative to collect that households’ allotted ITNs. Households were provided one ITN per sleeping space. Registered households that did not attend the community point distribution received ITNs from a CHW in a “mop-up” visit conducted within a day or two of the community point distribution. This visit was conducted separately from the hang-up visit, except in cases when it coincided with the household’s assigned hang-up day. Households that were not pre-registered but attended the distribution event received ITNs as available after all registered households had received their ITNs.

The second component of the intervention was a CHW hang-up visit, during which CHWs visited households to record how many ITNs from the distribution were found in the household and how many were already hanging, as well as to hang any remaining ITNs. While CHWs may have repeated standard malaria messages on their own initiative during these visits, this was not explicitly part of the intervention. Prior to the distribution, pre-registered households were stratified by the CHW who pre-registered them. Within each stratum, households were then randomised into one of five groups to receive hang-up visits at different intervals. CHWs visited households allocated to Group 1 at 1–3 days after the community point distribution, Group 2 at 5–7 days, Group 3 at 10–12 days, and Group 4 at 15–17 days. Group 5 did not receive a hang-up visit. These groups were constructed in order to examine the ITN self-installation rates by households over time and to assess the impact of CHW hang-up visits (Groups 1–4) on ITN retention and usage compared to those that did not receive a hang-up visit (Group 5).

### Definition and measurement of outcomes

The study focused on four primary outcomes. The first was household attendance at the community point distribution, measured as the percentage of registered households that collected ITNs at the distribution. The second outcome was the percentage of ITNs self-installed by households at different durations after the distribution. This was calculated as the percentage of distributed ITNs found hanging in the household at the time of the hang-up survey. Households that were randomly selected to not receive a hang-up visit were excluded from this outcome. The third outcome was the average household ITN retention ratio, defined as the average percentage of distributed ITNs that were self-reported by households to be present at the time of the follow-up surveys. The majority of these ITNs were also directly observed in the household by study staff (“verified”). Some ITNs were not observed either because the household member being interviewed did not have access to the rooms in which they were stored or because household members were unwilling to allow entry to study staff in the absence of the household head. The fourth and final outcome was ITN usage. Two metrics were used to approximate usage: 1) average percentage of sleeping spaces covered by an ITN per household and 2) average percentage of distributed ITNs hanging per household. For the self-installation, retention, and usage outcomes, CHWs and study staff were instructed to count and to ask about only those ITNs that were provided from the distribution. These ITNs were identified by their Permanet brand, blue colour, and by directly asking household representatives if the ITN was received at the recent distribution.

### Data collection and data quality

The study team for this evaluation consisted of IDinsight staff. Field managers are Zambian nationals who are permanent employees and manage the logistics and supervision of data collection for various IDinsight evaluations. Field officers were Zambian nationals hired as enumerators for this evaluation. They were trained by IDinsight field managers and higher level staff on data collection techniques, use of data collection devices, evaluation objectives and survey instruments, and fieldwork logistics.

At each community point distribution, study staff recorded data on attendance, number of ITNs distributed, and registration status. Household members signed or placed a thumbprint on a distribution register to verify that they had received the number of nets for which they had been pre-registered.

At each hang-up visit, CHWs recorded information on the number of ITNs found in the household, the number found hanging, and the number they hung to assess household self-installation rates. Household members signed or placed a thumbprint on the survey to confirm they had been visited by the CHW for the hang-up survey. Whenever possible, CHWs directly observed ITNs and sleeping spaces. Study staff members were present for approximately 25 % of CHW hang-up visits in each zone to ensure the accuracy and quality of these surveys. Additionally, 10 % of hang-up surveys (n = 41) were randomly selected for resurvey by study staff within 3 days of the initial hang-up visit. Of these 41 households, 90.2 % (n = 37) were available for a resurvey. All but one (3 %) of these resurveys recorded the same number of ITNs found in the households as the original hang-up survey.

Follow-up surveys at two different time points were conducted by study staff with all households included in the study (including households that did not receive a hang-up visit). In order to have results available ahead of the release of national recommendations for the 2014 ITN distribution, a follow-up survey was conducted within 7–11 weeks following the community point distribution to examine short-term retention and usage. To better understand medium-term retention and usage, households were also visited between 5 and 6 months using a nearly identical follow-up survey. Data for both of these visits were collected using Open Data Kit (ODK) on Samsung Galaxy Y© phones. Study staff collected information on household ITNs (both present and those no longer in the household), sleeping spaces, and other household information. Before conducting each follow-up survey, study staff obtained consent from households, via a signature or thumbprint on the consent form. As with the hang-up visit, study staff attempted to visually confirm all sleeping spaces and present ITNs.

For the 7–11 week follow-up, 5 % of households (n = 28) were re-visited by study staff to conduct back-check surveys for data quality assurance. These surveys had identical ITN ownership numbers to those recorded in the original survey for 82 % (n = 23) of households with back-check surveys. For the 5–6 month follow-up, 10 % of households (n = 56) were re-visited. Among these back-check surveys, 73 % (n = 41) of households had identical ITN ownership numbers to those recorded in the 5–6 month follow-up survey. Discrepancies can largely be attributed to differences in survey respondents and to movement of household members and ITNs. Comparisons of GPS data, household characteristics, and respondents’ surnames were also compared to verify the identity of households. There were no cases where inaccurate or fraudulent data were suspected after investigating these discrepancies.

### Sample size

Sample size calculations were made based on the ITN usage outcome. The original calculation planned to include a fourth community (Mwakapila) and used the following parameters: Assuming α = 0.05 (two-sided) and 84 % power, a sample of 662 households (440 in Groups 1–4 and 222 in Group 5/comparison group) would enable us to detect an increase in ITN usage from 60 to 72 %. The calculation assumed a 95 % plausibility interval of 30–80 % for the control group, and treated the household as the unit of randomisation.

Due to logistical constraints, only three communities were included in the study. An updated calculation using the above parameters and a sample of 528 households (the # of households receiving 7–11 week follow-up survey, 358 in Groups 1–4 and 170 in Group 5) would enable the study to detect an increase in ITN usage from 60 to 73 %.

All power calculations were done using Optimal Design (Optimal Design Co, Arlington Heights, Illinois, USA).

### Ethical approval

This study was approved by the ERES Converge Ethics Review Board in Lusaka, Zambia. Authority for the study implemented was obtained from Zambia’s Ministry of Health and Ministry of Community Development, Mother and Child Health.

### Statistical methods

The analysis of study data focused on understanding ITN self-installation, retention, and usage. All statistical analyses were done using Stata 12 (Stata Corp LP, College Station, Texas, USA). The percentage of distributed ITNs that were hung at the time of the CHW hang-up visit was calculated with 95 % confidence intervals (CI) and plotted as a bar graph. Self-installation rates between the groups were considered statistically significantly different if the 95 % CIs did not include unity.

Retention ratios were calculated for all ITNs, and average household retention ratios were compared between households that received a hang-up visit (groups 1–4) and those that did not (group 5) using a linear regression model with a Huber-White sandwich estimator for robust standard errors. Separate regressions were run using the 7–11 week outcomes and the 5–6 month outcomes. All models were adjusted for the same covariates. Straight line distance from the household to the distribution site was included as a covariate due to concern that households traveling from further away may be less likely to retain the ITNs received. The CHW who initially registered the household was included, since this was the variable on which the data were stratified for randomisation. The number of days between the community point distribution and the follow-up visit, the number of household members, and the method by which the household received its ITNs (mop-up visit versus at the community point distribution) were also included since these could impact household retention and usage.

Linear regression models adjusting for the same covariates were also used to compare the two measures of ITN usage (average percentage of ITNs hanging and average percentage of sleeping spaces covered) between households that received a hang-up visit and those that did not. Statistical significance for all regressions was defined as *p* value <0.05.

### Time-savings analysis

To enable estimation of the time savings of the community point distribution approach, the study authors assessed the personnel time used by various components of the community point distribution and hang-up visit activities including conducting a community point distribution, traveling between households, collecting ITNs from storage, assessing how many ITNs had been hung, and hanging ITNs. These measurements were used to model the projected time for community point and door-to-door distributions. A full description of inputs is included in Table 1.

**Table 1:** Inputs for human resource analysis

Inputs	Number	Units	Note
*Nets*			
HH per zonal distribution	198	households	Average # study HHs per zone^1^
Point distribution attendance rate	96 %	percent	Based on distribution event attendance^1^
Percentage HHs requiring mop-up visit	4 %	percent	Based on distribution event attendance^1^
Nets per household	2.7	nets	Average nets per household was 2.7^1^
Self-installation rate	74 %	percent	Mean self-installation rate 10–17 days after distribution
Number of nets to be distributed	5750,300	nets	4,810,300 from UNDP ITN Operational plan.^2^ TWG goal was 8,998,200, with 5,750,300 committed at time of plan publication
% of available ITNs to be distributed to rural areas	75 %	percent	60 % of Zambia population is rural (2012 figure, data.worldbank.org). Assume full coverage for rural area; 50 % coverage for urban areas
*Distances*			
Distance multiplier	1.5		Distances are measured using GPS coordinates. This accounts for the indirectness of roads
Straightline distance from clinic to community	5.3	km	Straightline average distance between point distribution and clinic was 5.3 km^1^
Travel distance from clinic to community	7.9	km	
*Times*			
Time for introductions	5	min	Introduction, entry permission, fetching hh head
Avg. travel time between HH	11.51	min	Mean travel-time walking from evaluation: 15.35.^1^ Speed calculated using average walking and cycling speeds
Burden of carrying nets (time multiplier)	1.25	multiplier	Transport w/ITNs and fetching ITNs from storage
Time to hang one net	10	min	Conservative estimate based on field obs
Workday Length	8	h	
Point distribution publicity CHW	1	Man days	Based on field observations^1^
Point distribution	8	h	Based on field observations^1^
Survey time	5	min	Basic data collection (all HH visits)
Averate trip time fetching nets	1.79	h	
*CHW*			
Number of CHWs allocated by UNDP	6945	CHW	50 nets a day/CHW and 14 days to complete^2^
CHWs day per supervisor day	22	CHW days	Assumption
CHWs per point distribution	4	CHWs	Based on field observations^1^
Average # of HHs to visit per day (D2D)	7	HHs	Accounts for time fetching nets
*Transport costs*			
Average walking speed	5.00	km/h	Avg walking speed
Average cycling speed	10.00	km/h	Avg cycling speed
Average speed carrying nets	8.00	km/h	Incorporating ITN carrying multiplier
Average # of nets distributing per day	18	nets	# of ITNs distributed per CHW per day (with time for one trip to storage to fetch nets)
Average net transport capacity	100	nets	Maximum # of nets a CHW can transport per trip (via bicycle)

^1^Demand Driven Evaluations for Development (3DE). February, 2014. ITNs 6-week follow-up Survey Data

^2^Zambia Ministry of Health, United Nations Development Program (UNDP), Churches Health Association of Zambia (CHAZ). 2014. Towards Universal Coverage of Long-Lasting Insecticidal Nets (LLINs) in Zambia: Operational Distribution Plan for the Year 2014

## Results

**Fig. 1:**
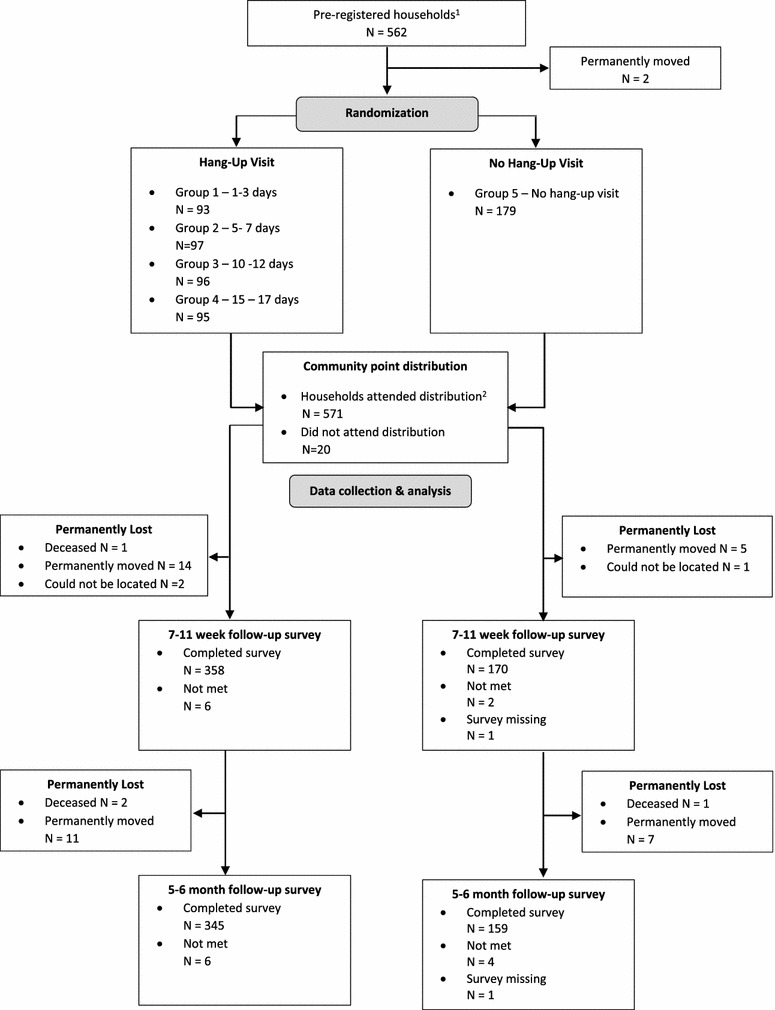
Flowchart of household participation. ^1^Two households were not on the NMCC pre-registration list, but they were added by community health workers prior to distribution. ^2^There were an additional 31 households that attended distribution but were not on the registration list

CHWs pre-registered 562 households. Two of these households permanently moved prior to distribution, leaving 560 households that were randomised to one of the five hang-up groups (Fig. 1). On average, households that were randomised to receive a hang-up visit were similar to households that did not receive a hang-up visit in household size, number of sleeping spaces, education level of the head of household, household distance to the clinic, and distance to the distribution site (Table 2). Households in each treatment group were also similarly distributed across zones. Of the 560 households included in the primary sample, 528 (94.3 %) were available for the 7–11 week survey and 504 (90.0 %) were available for the 5–6 month survey.

**Table 2:** Household characteristics by hang-up visit

Household characteristics	No hang-up visit (N = 179)		Hang-up visit (N = 381)	
	Mean	(SD)	Mean	(SD)
Sleeping spaces	2.76	(1.25)	2.72	(1.27)
Household size	5.18	(2.72)	5.11	(2.69)
Distance to clinic (km)	5.00	(4.17)	4.69	(3.91)
Distance to distribution site (km)	1.55	(1.28)	1.48	(1.21)

	N	(%)	N	(%)
*Zone*				
Lukwipa	74	(41.57)	156	(40.73)
Mukonka	61	(34.27)	149	(38.9)
Chipeketi	43	(24.16)	76	(20.37)
*Education level of household head*				
No formal education	22	(16.3)	54	(18.82)
Primary	84	(62.22)	171	(59.58)
Secondary	29	(21.48)	54	(18.82)
Professional training school	0		1	(0.35)
College/university	0		7	(2.44)

Missing values: Household size—N = 3; Distance to clinic—N = 27; Distance to distribution site—N = 27; Education level of HH head—N = 149

### Community point distribution attendance

The community point distributions occurred on November 14, 19, and 21, 2013. Of the 560 pre-registered households, 540 (96.4 %) attended distributions or sent a representative to receive ITNs. Of these 540 households in attendance, 55 % were represented by heads of households or their spouses while another 39 % were represented by other family members. Remaining households (6 %) were represented by non-family members who were primarily neighbours. The 20 pre-registered households that did not attend received a mop-up visit by a CHW. An additional 31 households (5.7 % of total attendees) attended the distribution but were missed in the original registration survey effort.

### Self-installation rate

**Fig. 2:**
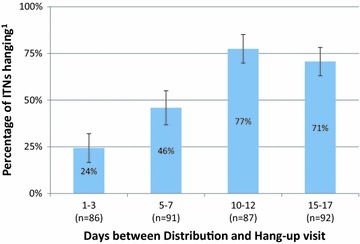
Self-installation rates among treatment groups in households visited at hang-up survey. ^1^ *Bars* represent 95 % confidence intervals

The self-installation rate generally increased over time with 24.3 % of ITNs [95 % CI 16.7 %, 31.9 %] hung at 1–3 days, 45.9 % [95 % CI 36.9, 54.9] hung at 5–7 days, 77.5 % [95 % CI 69.9 %, 85.0 %] hung at 10–12 days and 70.6 % [95 % CI 63.2 %, 78.1 %] hung at 15–17 days (Fig. 2). Differences between groups 1 and 2 and groups 2 and 3 indicated a statistically significant increase in self-installed ITNs over time. While there was a slight decrease in the self-installation rate between groups 3 and 4, the difference was not statistically significant, and subsequent follow-up surveys showed that the self-installation rate did not continue to decrease substantially over time.

### ITN retention

Seven to 11 weeks following the distribution, 90.2 % (n = 1312) of the 1454 ITNs distributed to the households available for this survey were still in the households, with 74.9 % (n = 1089) visually verified by research staff. On average, households had retained 88.8 % of their ITNs with a standard deviation (SD) of 23.2 %. Those that had received a CHW hang-up visit had retained 88.9 % (SD = 23.5 %) compared to an average retention ratio of 89.4 % (SD = 21.3 %) among those with no hang-up visit. After adjusting for covariates, this difference was negligible at 0.08 percentage points (p value = 0.97, 95 % CI −3.9, 4.1) (Table 3).

**Table 3:** Average outcomes by hang-up visit and multivariate linear regression results

Outcome	No hang-up visit			Hang-up visit			Adjusted difference^a^			
	N	Mean	(SD)	N	Mean	(SD)	N	Coeff	p value	95 % CI
*7*–*11* *weeks*										
Percentage of ITNs retained in household	170	89.36	(21.26)	358	88.94	(23.50)	517	0.08	0.97	[−3.92, 4.09]
Percentage of sleeping spaces covered	169	68.30	(37.98)	356	76.45	(33.41)	514	8.80	<0.01	[2.35, 15.25]
Percentage of ITNs hanging	170	60.19	(36.06)	357	68.16	(32.96)	516	8.83	<0.01	[2.56, 15.11]
*5*–*6* *months*										
Percentage of ITNs retained in household	157	86.26	(24.15)	340	86.43	(24.06)	494	1.68	0.46	[−2.78, 6.14]
Percentage of sleeping spaces covered	159	79.08	(35.66)	345	80.86	(33.95)	501	2.38	0.47	[−4.05, 8.81]
Percentage of ITNs hanging	159	61.87	(34.57)	345	63.76	(33.84)	501	2.97	0.34	[−3.18, 9.13]

^a^All models were adjusted for the straight line distance from the household to the distribution site, the CHW who initially registered the household, the number of days between the community point distribution and the follow-up visit, and the number of household members, and the method by which the household received its ITNs (mop-up visit versus at the community point distribution)

Five to six months after the distribution, households had retained a total of 85.7 % (n = 1196) of the 1395 ITNs distributed to households available for the 5–6 month survey, with 79.4 % (n = 1108) visually verified. On average, households had retained 86.1 % (SD = 24.4 %) of their nets. Households that received a CHW visit had retained an average of 86.4 % (SD = 24.0 %) compared to an average of 86.3 % (SD = 24.2 %) among households in group 5. After adjusting for covariates, the average percentage of ITNs retained among households who had received a CHW hang-up visit was 1.7 percentage points higher than among households that had not received a visit (p value = 0.46, 95 % CI −2.8, 6.1).

Common reasons that were given by households for not having the ITNs in the household were that they were used elsewhere by household members, they were given or sold to another household, or they were kept by the representative who attended the distribution on behalf of the household.

### ITN usage

#### Sleeping space coverage

Study households had an average of 2.7 (SD = 1.3) sleeping spaces. During the 7–11 week follow-up, the average percentage of sleeping spaces covered per household was 73.8 % (SD = 35.1 %). Households that had not received a hang-up visit had an average of 68.3 % (SD = 38.0 %) sleeping spaces covered while households that had received a hang-up visit had an average of 76.5 % (SD = 33.4 %) sleeping spaces covered. After adjusting for covariates, the average percentage of sleeping spaces covered was 8.8 percentage points higher (p value < 0.01, 95 % CI 2.4, 15.3) among those with a hang-up visit (Table 3).

Overall, sleeping space coverage was greater in the 5–6 month survey than in the 7–11 week survey, which is likely explained by a difference in seasons or the end of the school year. The 5–6 month follow-up found an average of 80.3 % (SD = 34.5 %) of sleeping spaces covered per household, with an average of 79.1 % (SD = 35.7 %) sleeping spaces covered among households with no hang-up visit and an average of 80.9 % (SD = 33.9 %) sleeping spaces covered in the households with a hang-up visit. The difference between the two groups was more modest and statistically insignificant in the 5–6 month follow-up; households that had received the hang-up visit had an average of 2.4 percentage points more sleeping spaces covered (p value = 0.47, 95 % CI −4.1, 8.8).

### ITNs hanging

On average, households had 65.6 % (SD = 34.1 %) of their distributed ITNs hanging 7–11 weeks after distribution. This average was slightly lower among households that had not received a hang-up visit (mean = 60.2 %, SD = 36.1 %) than among households that had received a hang-up visit (mean = 68.2 %, SD = 33.0 %). After adjusting for covariates, a hang-up visit was associated with an 8.8 percentage point increase in the percentage of hanging ITNs (p value < 0.01, 95 % CI 2.6, 15.1) (Table 3).

At the 5–6 month follow-up, households had an average of 63.1 % (SD = 34.0 %) of ITNs hanging, with 61.9 % (SD = 34.6 %) in the group that did not receive a hang-up visit compared to 63.8 % (SD = 33.8 %) in the hang-up group. The adjusted difference was 3.0 percentage points (p value = 0.34, 95 % CI −3.2, 9.1). There was no significant relationship between the percentage of ITNs reported hanging and the treatment groups in the 5–6 week follow-up period.

### Time-savings analysis

**Fig. 3:**
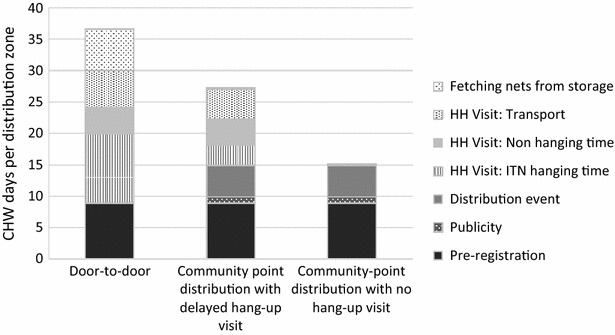
Time requirements by ITN distribution method

According to a model based on evaluation findings, the community point distribution method with CHW hang-up visits reduced the time required for an ITN distribution by 25 % when compared to door-to-door distribution method (Fig. 3). Furthermore, the community point distribution method without hang-up visits (the control group in this study) reduced the time required for an ITN distribution by 59 % when compared to the door-to-door distribution method. The greatest time-savings with the community point distribution are the ITN hanging time and the time spent collecting nets from storage.

## Discussion

This evaluation revealed that community point distributions can achieve high attendance rates as well as high short- and medium-term ITN retention and usage levels in rural Zambia. This finding of 96 % attendance is similar to or higher than other mass distributions. A cross-sectional survey of community point distributions in Zanzibar, Tanzania found that 84 %-97 % of registered households attended distributions [19], while another analysis of point distributions in northwest Tanzania found an attendance rate of 84 % [14]. These studies contain few details about how the campaign sites were chosen. Anecdotal evidence in Zambia suggested that point distribution attendance suffered when RHCs were used as distribution points due to the long travel distances to reach these sites (up to a full day of travel each way in some cases). The community point distributions included in this study were conducted at the neighbourhood zone level, one level below the RHC level, which limited travel time to a maximum of 4 h.

The majority of distributed ITNs in the study were self-installed within the first 10 days following the community point distributions. This suggests that significant CHW time-savings can potentially be achieved by delaying hang-up visits by at least 10 days, when more than 70 % of ITNs were self-installed in households. A study from Togo also found that 58 % of campaign ITNs were hung by the time the CHW visited immediately following a campaign [17], and a study from Uganda found that 55.7 % of households had all nets hanging at the time of a visit immediately after a mass campaign [18]. This suggests that households will self-install ITNs that they expect to use.

Overall, the households included in this study demonstrated high retention ratios of 89 % at 7–11 weeks following distribution and 86 % at 5–6 months following distribution. Usage was also high with 74 % sleeping space coverage at 7–11 weeks and 80 % sleeping space coverage at 5–6 months. These rates are similar to the benchmarks set by government stakeholders based on retention and usage following a door-to-door campaign in Luapula Province, which found 96 % ITN retention with 79 % of sleeping spaces covered after 8 weeks (STEPS OVC, personal communication, 2013). This suggests that community point distributions could be a viable alternative to the standard door-to-door distributions in Zambia. Additionally, percentage of sleeping spaces covered could be a conservative estimate of usage. Some households are very small and, therefore, take down the ITN during the day so it is not in the way. Further, with movement of household members, especially during the harvest or during the school year, some sleeping spaces may not have been used at the time of the survey.

According to the second measure of usage, average percentage of distributed ITNs found hanging, households had an average of 63 % of ITNs hanging at 7–11 weeks and 66 % at 5–6 months. This measure is lower than the percentage of sleeping spaces covered. The number of ITNs a household received during distribution was based on the number of sleeping spaces recorded during the pre-registration activities. It is possible that more sleeping spaces were pre-registered than actually existed. On the other hand, though the study team asked about and tried to verify ITNs that came from the distribution (based on the colour and on the brand), it was possible that respondents were still reporting that a sleeping space was covered with a non-distribution ITN.

While CHW hang-up visits appeared to have a short-term impact on the two measures of ITN usage, this impact was not detected in the 5–6 month survey, nor was this effect detected on ITN retention. A clustered RCT from Uganda tested the impacts of a single CHW visit 4 months after distribution and two hang-up visits at 4 and 7 months after distribution on ITN usage. These visits coupled hang-up activities with messaging about the importance of malaria prevention and ITN usage. Neither intervention arm had a statistically significantly different usage rate compared to the control group of no CHW visits, and all three arms followed similar trends in usage over time [18]. Another clustered RCT from Togo found a modest impact of door-to-door visits on ITN usage, but again, these visits were coupled with health messaging. The findings from this study suggest that households may be likely to hang the ITNs that they will use on their own, and while CHW hang-up visits may achieve immediate increases in usage, eventually households will take down ITNs that they do not use. It is possible that these results would have been sustained if the CHWs in this study had also reinforced messaging around malaria prevention and ITN usage and care.

The time-savings analysis indicated that a community point distribution with delayed hang-up visit can reduce the time it takes to distribute nets by 25 % and a community point distribution with no hang-up visit can reduce the time it takes by 59 %. Coupled with the fact that community point distributions can achieve similar levels of retention and usage as door-to-door campaigns in Zambia, community point distributions could be a more cost-effective alternative to labour-intensive door-to-door campaigns. Settings where community point distributions may achieve the highest gains are areas where communities are far away from the storage facility, areas in which inhabitants are likely to be away from home when visited by CHWs, areas without established CHWs who can deliver door-to-door, and contexts with strong community ties to ensure a well-monitored community point distribution and effective community sensitization to ensure high attendance. The time-savings analysis was done from the government perspective, since there may be opportunity costs to government programmes for CHW time. Community members’ time was not included in the model, but that should also be considered when making policy decisions.

Results from this evaluation, along with operational plans to support implementation of community point distributions, were shared with the MOH, NMCC, and donors supporting ITN distribution in Zambia. As a result of these findings, community point distributions were included in national guidelines as a viable alternative to door-to-door distributions in districts that were deemed appropriate for these activities. Operational plans relied heavily on implementation experiences and lessons learned during this study.

### Limitations

This evaluation was completed in rural Rufunsa district of Zambia, and, therefore, does not purport to explore the effectiveness of community point distribution in urban, peri-urban, or extremely remote settings. Additionally, these results might not be generalizable to rural settings that fit a different geographic or population density profile. Further, meaningful malaria sensitization activities (including radio messages) had been conducted in Rufunsa prior to the evaluation. Community point distribution dynamics may differ if conducted in areas with less prior knowledge about malaria and ITNs.

The evaluation design does not directly compare community point distribution with the door-to-door distribution method. This direct comparison would have required randomisation at the zone level, which is expensive and was deemed unnecessary by MOH officials for informing a policy recommendation and decision. Instead, the study made use of a recent door-to-door distribution campaign and national malaria figures to draw comparisons between outcomes, though the study team did not have control of the data collected for these campaigns.

Given the obvious difficulty of monitoring actual ITN usage at night, this study defines usage as ITNs hanging in the household at the time of the survey. This may be a more conservative proxy than self-reported “usage” at night, as many respondents are aware that they should use the ITNs and are likely to give the “correct” answer. While some ITNs that are hanging may not actually be used at night, the study team also encountered respondents who reported using ITNs at night then taking them down during the day in order to free up space in the home.

Because randomisation occurred at the household level and not the village level, there is a possibility that self-installation rates could be artificially high due to households observing CHW visits to other nearby households. This might imply that CHW visits are pre-requisites for replicating the achieved self-installation rates at scale. Similarly, retention and spaces covered could be affected by households hearing or seeing surveyors in the area and wanting to exhibit the correct behaviour. While this is a possibility, distances between households were generally so great (~15 min walk from each other) that these effects were likely to be minimal. Finally, CHWs, themselves, recorded the self-installation outcomes during their hang-up visit. Though the study team accompanied 25 % of these visits and back-checks revealed high agreement with the CHW-recorded data, it is still possible that CHWs did not record data correctly.

## Conclusions

Community point distributions can be an effective and efficient means to distributing a high quantity of ITNs quickly in rural Zambia. Delaying CHW hang-up visits by 10 days or more could reduce the CHW ITN hanging workload by more than 70 %. Additionally, CHW visits may impact ITN usage in the short-term, but these impacts were not evident in the medium-term. Therefore, in time- and resource-constrained contexts, community point distributions without CHW follow-up to hang ITNs can yield additional time and cost savings.
